# Efficacy and safety of CD19-specific CAR-T cell-based therapy in secondary central nervous system lymphoma

**DOI:** 10.3389/fimmu.2022.965224

**Published:** 2022-08-19

**Authors:** Huanxin Zhang, Zhiling Yan, Ying Wang, Yuekun Qi, Yongxian Hu, Ping Li, Jiang Cao, Meng Zhang, Xia Xiao, Ming Shi, Jieyun Xia, Sha Ma, Jianlin Qiao, Hujun Li, Bin Pan, Kunming Qi, Hai Cheng, Haiying Sun, Feng Zhu, Wei Sang, Depeng Li, Zhenyu Li, Junnian Zheng, Mingfeng Zhao, Aibin Liang, He Huang, Kailin Xu

**Affiliations:** ^1^ Department of Hematology, The Affiliated Hospital of Xuzhou Medical University, Xuzhou, China; ^2^ Blood Diseases Institute, Xuzhou Medical University, Xuzhou, China; ^3^ Bone Marrow Transplantation Center, The First Affiliated Hospital, School of Medicine, Zhejiang University, Hangzhou, China; ^4^ Department of Hematology, Tongji Hospital of Tongji University, Shanghai, China; ^5^ Department of hematology, Tianjin First Central Hospital, Tianjin, China; ^6^ Cancer Institute, Xuzhou Medical University, Xuzhou, China; ^7^ Center of Clinical Oncology, The Affiliated Hospital of Xuzhou Medical University, Xuzhou, China

**Keywords:** secondary central nervous system lymphoma, chimeric antigen receptor t cell, relapsed/refractory, immunotherapy, immune effector cell-associated neurotoxicity syndrome

## Abstract

Encouraging response has been achieved in relapsed/refractory (R/R) B-cell lymphoma treated by chimeric antigen receptor T (CAR-T) cells. The efficacy and safety of CAR-T cells in central nervous system lymphoma (CNSL) are still elusive. Here, we retrospectively analyzed 15 patients with R/R secondary CNSL receiving CD19-specific CAR-T cell-based therapy. The patients were infused with CD19, CD19/CD20 or CD19/CD22 CAR-T cells following a conditioning regimen of cyclophosphamide and fludarabine. The overall response rate was 73.3% (11/15), including 9 (60%) with complete remission (CR) and 2 (13.3%) with partial remission (PR). During a median follow-up of 12 months, the median progression-free survival (PFS) was 4 months, and the median overall survival (OS) was 9 months. Of 12 patients with systemic tumor infiltration, 7 (58.3%) achieved CR in CNS, and 5 (41.7%) achieved CR both systemically and in CNS. Median DOR for CNS and systemic disease were 8 and 4 months, respectively. At the end point of observation, of the 7 patients achieved CNS disease CR, one was still alive with sustained CR of CNS disease and systemic disease. The other 6 died of systemic progression. Of the 15 patients, 11 (73.3%) experienced grades 1-2 CRS, and no patient had grades 3-4 CRS. Immune effector cell-associated neurotoxicity syndrome (ICANS) occurred in 3 (20%) patients, including 1 (6.6%) with grade 4 ICANS. All the CRS or ICANS were manageable. The CD19-specific CAR-T cell-based therapy appeared to be a promising therapeutic approach in secondary CNSL, based on its antitumor effects and an acceptable side effect profile, meanwhile more strategies are needed to maintain the response.

## Introduction

Secondary central nervous system lymphoma (CNSL) refers to secondary involvement of the neuroaxis by systemic disease, which often indicates aggressive disease with unfavorable survival comparing with systemic disease without CNS involvement (1, 2). Median survival was four months after diagnosis of secondary CNSL (3). The lack of effective treatments for these patients represents a critical unmet clinical need. Prospective phase II and III studies have confirmed the efficacy of high-dose antimetabolites and consolidative therapy, including whole-brain radiotherapy (WBRT) or high-dose therapy (HDT) with autologous stem cell transplantation (ASCT), in patients with primary CNSL (4–6). However, there are no such data for patients with secondary CNSL. Only few reports support the use of analogous treatment strategies in secondary CNSL, but the feasibility of HDT/ASCT is limited for these patients due to failure of salvage treatment, toxicity and unsuccessful stem cell harvest (7, 8).

CAR-T cell therapy, as a novel immunotherapy approach, significantly improves the outcome of relapsed/refractory (R/R) B-cell non-Hodgkin lymphoma (9–11). Because of concerns for CAR T-cell-related neurotoxicity (NT), patients with active CNS involvement were not included in most pivotal studies. In 2017, Abramson et al. reported the efficacy of CD19-specific CAR-T cells in secondary CNS diffuse large-B-cell lymphoma (12), indicating that CNSL is not an absolute contraindication for CAR-T cell therapy. Subsequently, some studies reported the efficacy of CAR-T cell therapy in primary and secondary CNSL, and adverse events were controllable (12–16). However, most of these studies were small clinical analyses or case reports. More clinical cases are needed to further confirm the efficacy and safety. Here, we analyzed response, toxicity and feasibility of CD19-specific CAR-T cell-based therapy in R/R secondary CNSL.

## Materials and methods

### Study design

15 patients with R/R secondary CNSL disease were enrolled between July 1, 2017, and August 1, 2021. Lymphomas were diagnosed according to World Health Organization (WHO) Classification standards (17). This study has been approved by the respective ethics committee and registered with the Chinese Clinical Trial Registration Center (ChiCTR-OIC-16008291, ChiCTR1800015575) and ClinicalTrials.gov (NCT03207178). Informed consent was obtained from all participants, in compliance with the Declaration of Helsinki.

### CAR-T cell manufacture

The preparation process of CAR-T cells has been previously described (18–20). The costimulatory molecules are 4-1BB. The lentivirus vector for CD19, CD20 or CD22 CAR was established with co-stimulating molecules, CD8 transmembrane region, CD8 hinge, and CD3 zeta signaling domain. CD3^+^ T cells isolated from peripheral blood mononuclear cells (PBMCs) were transfected by packaged lentivirus.

### Procedures

PBMCs from patients eligible for the clinical trial were collected to prepare CAR-T cells. During CAR-T cell preparation, bridging therapy was allowed if the patient’s primary disease is progressing too rapidly. Patients received three daily doses of fludarabine 30 mg/m² and one dose of cyclophosphamide 750 mg/m² before CAR T cell infusion. Infusion patterns included isolated infusion of anti-CD19 CAR-T cells, sequential infusion of anti-CD19/20, and infusion of anti-CD19/22 dual-targeted CAR-T cells.

### Efficacy and toxicity assessment

CNS and systemic responses were assessed according to the International PCNSL Collaborative Group Response Criteria (21) and Lugano Response Criteria of B cell lymphoma (22), respectively. Cytokine release syndrome (CRS) and immune effector cell-associated neurotoxicity syndrome (ICANS) were evaluated and graded according to the ASTCT Consensus Criteria (23). Other adverse events (AEs) were graded using the National Cancer Institute Common Terminology Criteria for Adverse Events, version 4.03 (24). According to the patients’ tolerance and the severity of CRS and NEs, the intervention therapies of corticosteroids and tocilizumab were given (25, 26).

### Statistical analysis

PFS was defined as the time from first infusion of CAR-T cells to progression of disease (CNS or Systemic) or death. Duration of remission (DOR) was defined as time from CR/PR to relapse or death without documented relapse. OS was defined as the time from first infusion of CAR-T cells to the date of death. The Clopper-Pearson 95% confidence interval (CI) and Fisher’s Exact test were used to analyze the classification variables. Kaplan-Meier methodology was used to estimate the medians for PFS and OS survival. Log-rank test was used to compared the survival of different groups. Follow-up periods were calculated using the reverse Kaplan-Meier method. SPSS 25 statistical analysis software was used for analysis, and p value less than 0.05 was considered as significant difference.

## Results

### Patient baseline characteristics

In total, 15 secondary CNSL patients from 4 centers were included in our retrospective study. Patient characteristics are presented in **Table 1**. The median age at CAR-T cell infusion was 51 years (range, 31- 66 years) with 46.7% of the patients aged over 60 years. These patients comprised 11 males and 4 females. Twelve of the 15 secondary CNSL patients had systemic disease, and 3 patients had only isolated CNSL. Eight patients had primary refractory disease and 7 had relapsed disease. The median of therapy lines was 3 (range, 2–17) before enrolment and 2 (10.5%) patients had received ASCT. The patient’s Karnofsky Performance score (KPS), pathologic subtypes, previous treatment regimens and localization of CNS disease are presented in **Table 1**. Double - or triple - hit rearrangements was detected in 3 (20%) patients, and P53 gene mutation in 2 (13.3%) patients. All patients underwent an age-adjusted international prognostic index (aa-IPI) score, with 6 patients scoring 0-2 and 9 patients scoring 3-4. Eleven patients (73.3%) were infused with anti-CD19 CAR-T cells, two (13.3%) with CD19/CD20 CAR-T cells, and 2 (13.3%) with CD19/CD22 CAR-T cells (**Table 1**).

**Table 1:** Clinical Characteristics at Baseline.

Characteristic	Patients (N=15)	Characteristic	Patients (N=15)
**Median age(range)**	51(31-66)	**Pathologic subtype**	
Age ≧ 60 yr	7	DLBCL	13
**Gender**		PMBCL	1
M	11	Burkitt	1
F	4	**localization of CNS disease**	
**Systemic disease**		Parenchymal	10
Yes	12	leptomeningeal	0
No	3	ocular	3
**Primary refractory or relapse**		CSF infiltration	6
Primary refractory	8	**aaIPI score**	
Relapse	7	0-2	6
**Prior lines of therapy**		3-4	9
2-3	8	**Double - or triple - hit rearrangement:Myc plus BCL2, BCL6, or both - no**	3
≥ 4	7	**P53 mutation**	2
**Previous autologous hematopoietic stem-cell transplantation**	2	**Bridging treatment**	
**Karnofsky Performance score**		Yes	8
20-40	2	No	7
50-60	4	**Infused cells**	
70-80	5	CAR19	11
90-100	4	Sequential CAR19/20	2
**Previous therapies**		CAR19/22	2
Rituximab	15	Average dose (*10^6/kg)	
high-dose MTX	4	<5	9
high-dose Ara-C	3	5∼10	2
Ibrutinib	6	≥10	4
Temozolomide	1		
Lenalidomide	5		
intrathecal injection	10		
Whole brain radiotherapy	5		

aaIPI, age-adjusted international prognostic index; DLBCL, diffuse large B-cell lymphoma; PMBCL, primary mediastinal large B-cell lymphoma.

### Response of the CNS disease

At the month 3 assessment, the ORR of CNS disease in the 15 secondary CNSL was 73.3% (11/15), including 9 (60.0%) patients having complete remission (CR), 2 (13.3%) partial remission (PR). Among the patients with CNS disease response, the median time for clinical symptoms of CNS (including headache, blurred vision, tinnitus, facial paralysis, confusion, etc.) to begin to be improved was 7.5 days (range, 3-14 days). The median time to the best response was 30 days (range, 8-116 days) based on cerebrospinal fluid testing and imaging evaluation.

All the baseline characteristics of the patients did not significantly affect the ORR, including age, gender, disease status (systemic + CNS or isolated CNS), disease nature of CNSL (relapsed or refractory), IPI score, CSF infiltration, types of CNS involvement (single lesion or multiple lesion), high-risk genotype (double/triple hit or TP53 mutation), bridging therapy, and infusion cell dose (**Supplementary Table 1**).

The patients’ previous treatment regimens, including intrathecal injection, HD-MTX, high-dose Ara-C, ibrutinib, lenalidomide, and craniocerebral radiotherapy, had no significant effect on the ORR (**Supplementary Table 2**).

### Survival

**Figure 1:**
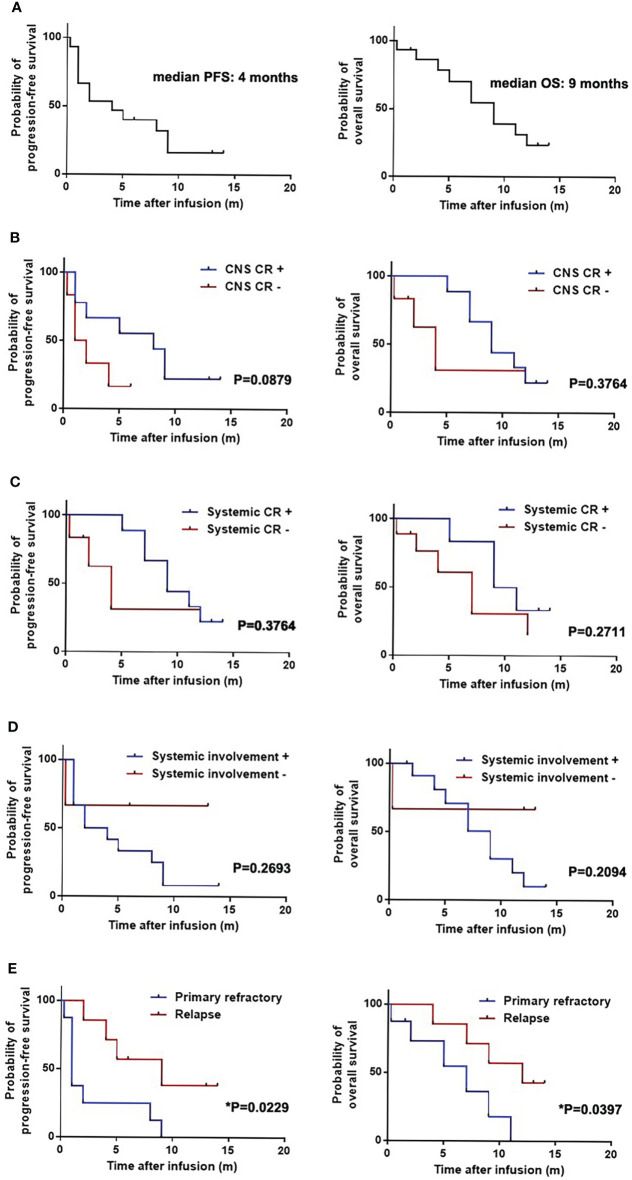
Progression-free survival (PFS) and overall survival (OS) of patients with secondary central nervous system lymphoma (CNSL) after CD19-specific CAR-T cell-based therapy. **(A)** PFS and OS of 15 patients with secondary central nervous system lymphoma (CNSL) after CD19-specific CAR-T cell-based therapy. **(B)** PFS and OS of secondary CNSL patients with or without central nervous system (CNS) disease complete remission (CR) after CD19-specific CAR-T cell-based therapy. **(C)** PFS and OS of secondary CNSL patients with or without systemic disease CR after CD19-specific CAR-T cell-based therapy. **(D)** PFS and OS of secondary CNSL patients with systemic involvement or not after CD19-specific CAR-T cell-based therapy. **(E)** PFS and OS of secondary CNSL patients having primary refractory lymphoma or relapsed lymphoma.

Fifteen patients were followed up for a median of 12 months (range, 0.25-22 months). The median PFS and OS was 4 months and 9 months, respectively (**Figure 1A**). Six-month OS and PFS were 70.5% and 40%, and 12-month OS and PFS were 23.5% and 16%, respectively.

Median PFS and OS in the patients with CNS disease CR were 8 and 9 months, respectively, which were 1.5 and 4 months in the patients without CNS CR (**Figure 1B**). Median PFS and OS in the patients with systemic and CNS disease CR were 9 and 10 months, respectively, which were 4 and 7 months in the patients without systemic and CNS CR (**Figure 1C**). Median PFS and OS in patients with systemic involvement were 3 and 9 months, however, which were not reached in patients without systemic involvement (**Figure 1D**). The patients with primary refractory lymphoma had shorter PFS than those with relapsed lymphoma (1 month vs 9 months, *p*=0.0229). Shorter OS was observed in the primary refractory lymphoma patients compared to the relapsed lymphoma patients (7 months vs 12 months, *p*=0.0397) (**Figure 1E**). Other baseline characteristics including age, gender, IPI score, CSF infiltration, types of CNS involvement (single lesion or multiple lesion), high-risk genotype (double/triple hit or TP53 mutation), prior ASCT, bridging treatment and infusion cell dose had no significant effect on PFS and OS (**Supplementary Table 3**). The previous treatment to the patients had no significant effect on the PFS and OS, which was similar to the effect to the ORR (**Supplementary Table 4**).

### The therapeutic response of patients with systemic infiltration

**Figure 2:**
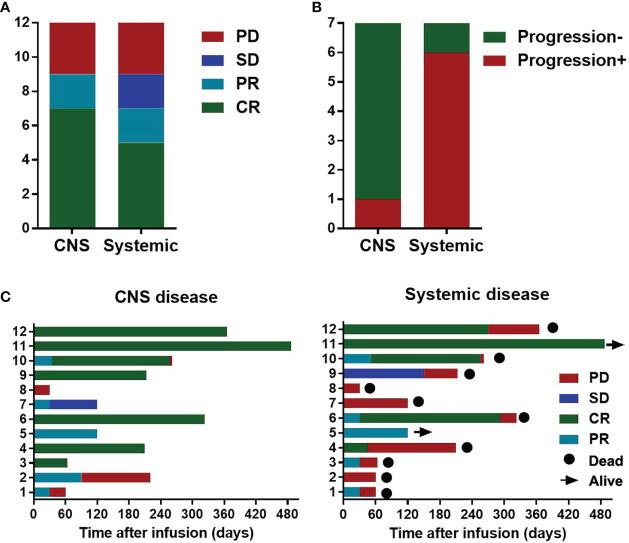
Disease response and survival of the 12 secondary Central Nervous System Lymphoma (CNSL) patients with systemic involvement. **(A)** Responses of the CNS and systemic disease after CAR-T cell infusion in the 12 secondary CNSL patients with systemic involvement; **(B)** Disease status at the data cutoff date for 7 patients with central nervous system disease complete remission (CR). **(C)** Disease status and survival of the 12 patients with secondary CNSL after CAR-T cell infusion.

Twelve patients had systemic lymphoma infiltration, including lymphadenectasis, liver infiltration, multiple skin and soft tissue infiltration, lung infiltration, bone marrow infiltration. At the time of CAR-T cell infusion, all patients had stage IV disease, and 8 patients had B symptoms. CNS disease CR was observed in 7 (58.3%) patients of the 12 patients, PR in 2/12 (16.7%), and progressive disease (PD) in 3/12 (25%). Systemic disease CR was achieved in 5 (41.7%) of the 12 patients, PR in 2/12 (16.7%), and stable disease (SD) or PD in 5/12 (41.7%) (**Figure 2A**). All the patients with systemic disease CR achieved CNS disease CR. At the end of observation, of the 7 patients achieving CNS disease CR, one was still alive with sustained CR of CNS disease and systemic disease. The other 6 died of systemic progression, with one suffered from relapse of CNS disease complicated with hemophagocytic syndrome (**Figure 2B**). The median DOR for CNS and systemic disease were 8 and 4 months, respectively. The disease status and survival of the 12 patients was shown in **Figure 2C**.

### Side effects

Eleven of the 15 patients had grade 1-2 CRS (73.3%), and no grade 3-4 CRS was observed. The median peak temperature of the 11 patients is 39°C(range, 37.6-40.2°C). Cytopenia occurred in 12 patients, including 10 patients with grade 3-4 cytopenia. Three patients had grade 1 gastrointestinal reactions, including diarrhea, constipation and vomiting. Four patients had grade 1 liver injury, 2 grade 1 renal injury, and 3 grade 1-2 cardiac injury. Three patients had grade 1 mucositis. Of the 11 patients with CRS, one patient was treated with tocilizumab, and the other received symptomatic and supportive treatment. No patient died of CRS. Three patients had ICANS (20%), including 2 grade 1 ICANS and 1 grade 4 ICANS. ICANS in 3 patients occurred at 12 h, 12 h and 16 h after CRS onset. CRS onset was defined by the time of their first fever after cell infusion which was 2 days, 2 days and 5 days, respectively. Symptoms of ICANS included headache, disorientation, convulsions, decreased computational ability, memory loss, insomnia, drowsiness, and seizures. The patient with grade 4 ICANS was treated with corticosteroid, and the other intervention including cranial pressure lowering, sedation and symptomatic support therapy. All the neurological events disappeared by above treatments and no patients had residual neurological impairment.

## Discussion

R/R secondary CNSL treatment is still a dilemma in the B-cell lymphoma therapy, although chemotherapy, cranial irradiation, and ASCT are all modalities that can be incorporated into the management of CNSL (7, 27). In the present study, we reported the efficacy, toxicity, and clinical feasibility of CD19-specific CAR-T cell-based therapy in 15 R/R secondary CNSL, of which, twelve had high systemic tumor burdon.

In our study, CD19-specific CAR-T cell-based therapy for R/R secondary CNSL resulted in a CNS ORR of 73.3%, and a CR rate of 60%, which were similar to the response of Tisagenlecleucel, Axicabtagene cells in the previous reports (13, 28, 29), and the sequential CD19/CD22 CAR-T cell immumotherapy following ASCT (30).

To our knowledge, there is no large-scale prospective study to clarify the PFS and OS of secondary CNSL patients treated with CAR-T cells. Our retrospective study showed that the PFS and OS were not perfect, the shorter remission might warrant consolidative strategies after CAR-T cell therapy, which is similar to the results observed in the previous studies (14, 15). CAR-T cell therapy combined with ASCT for CNSL appears to have encouraging long-term efficacy with relatively manageable side effects in the study by Wu et al; However, the timing and regimen of subsequent consolidation therapy for patients with high systemic tumor burden still need to be explored (30). In this study, the PFS and OS of patients with primary refractory lymphoma was worse than the patients with relapsed lymphoma. This might be related to the difference of biological characteristics of the patients. PFS and OS appeared to be better in patients with isolated CNS involvement than in patients with systemic involvement, probably because systemic disease progression accounted for the majority of deaths in cases with combined CNS involvement. From the limited data in the text, we speculated that without achieving CNS CR after CAR-T cell infusion, the primary refractory disease state, and combined with systemic disease might be potential risk factors for PFS and OS.

Rates of CR with conventional therapy for both systemic and CNS disease among patients with synchronous recurrence are low, ranging from 16-22%, and durable remissions are rare (7, 27). Compared with the conventional therapy, CD19-sepecific CAR-T cell-based therapy seemed to yield better CR rate of CNS disease (58.3%) and systemic disease (41.7%) in the 12 patients with secondary CNSL with systemic involvement. In our study, CNS disease seemed to have a longer DOR compared with the systemic disease (8 m vs 4m). We supposed that the CNS response may be similar or better than that of the systemic disease with the same CAR-T product and the same therapeutic target.

Non-Hodgkin lymphoma with Double-hit or triple-hit usually has a poor prognosis, as well as the mutation or deletion of p53 gene (31, 32). However, our research had not shown the influence of double-hit/triple-hit/P53 on the outcome of patients, which might be related to the small number of patients. While, it is also possible that CAR-T cell therapy could overcome the influence of these factors on the efficacy.

CRS is the most common complication of CAR-T cell therapy, and severe CRS may be life-threatening (25, 26). The incidence of grade 3 or worse CRS in lymphoma patients treated with CAR-T cells has been reported to be about 2-13% (9–11). In this study, no severe CRS was observed, and all the CRS were relieved after symptomatic support treatment, indicating that CD19-specific CAR-T cell-based therapy was relatively safe in treating secondary CNSL.

Neurotoxicity is a common complication following CAR-T cell therapy, and severe neurotoxicity has been associated with decreased survival after CAR-T cell therapy (25). According to previous reports, the median onset of neurologic events occurs on 4-5 days after CAR-T cell infusion. It can be concurrent with CRS, following resolution of CRS or occur alone (23, 25, 33). In B-cell non-Hodgkin’s lymphoma treated with CAR-T cells, the incidence of ICANS is 30-65%. The results of TRANSCEND NHL 001 preliminarily suggest that the incidence of ICANS of CNSL patients was not higher than that of ordinary lymphoma patients (9). In this study, there was no increase in the incidence or severity of ICANS compared with previous CAR-T cell therapy for systemic lymphoma, suggesting that CNS involvement should not be a limitation to CAR-T cell therapy. However, the onset time of ICANS in this study was relatively earlier compared with that in the systemic lymphoma. Due to the small number of cases in this study, the onset time of neurotoxicity needs more research to confirm.

Relapse or progression of CNSL after CAR-T therapy is one of the major challenges to be addressed in the future. The reasons for this restriction include limited CAR-T activity, loss of target antigen, and tumor microenvironment, etc (34). Combination with PD-1 blocking antibody (35) or immunomodulatory drugs (36, 37) could improve the CAR-T cells’ function and survival. Radiotherapy and hematopoietic stem cell transplantation could also be used in combination with CAR-T cells to enhance and consolidate the efficacy of CAR-T cells (30, 38). More exploration is needed to maintain response to CAR-T cell therapy.

## Conclusion

In conclusion, our study showed that the CD19-specific CAR-T cell-based therapy appeared as a promising therapeutic approach in secondary CNSL, based on its antitumor effects and acceptable side effects. Meanwhile further means are needed to maintain response to the disease. In secondary CNSL patients complicated with systemic tumor infiltration, the CNS disease might have a better response than systemic diseases.

## Limitations

Our findings are limited by the retrospective nature of the analyses, small number of cases and non-uniform CAR-T cells types. In the future, we plan to conduct with a prospective clinical study to further clarify the efficacy and safety of CAR T-cell therapy for secondary CNSL, as well as the selection and application timing of subsequent maintenance therapy. Further studies are warranted.

## Data availability statement

The raw data supporting the conclusions of this article will be made available by the authors, without undue reservation.

## Ethics statement

The studies involving human participants were reviewed and approved by Affiliated Hospital of Xuzhou Medical University; First Affiliated Hospital of Zhejiang University; Tongji Hospital of Tongji University ;Tianjin First Central Hospital. The patients/participants provided their written informed consent to participate in this study.

## Author contributions

KX, HH, AL, MeZ, MiZ, JZ, ZL designed the research. All investigators and their respective research teams recruited and followed up the patient. HZ, ZY, YW, YQ collected and analyzed research data. HZ, ZY wrote and edited the manuscript. All authors were involved at each stage of manuscript preparation and approved the final version. All authors contributed to the article and approved the submitted version.

## Funding

The authors would like to thank the financial support provided by the National Natural Science Foundation of China (grant No. 81871263, 81930005, Kailin Xu), Scientific Research Project of Jiangsu Commission of Health (grant No. Q201506, Huanxin Zhang).

## Conflict of interest

The authors declare that the research was conducted in the absence of any commercial or financial relationships that could be construed as a potential conflict of interest.

## Publisher’s note

All claims expressed in this article are solely those of the authors and do not necessarily represent those of their affiliated organizations, or those of the publisher, the editors and the reviewers. Any product that may be evaluated in this article, or claim that may be made by its manufacturer, is not guaranteed or endorsed by the publisher.
