# Supplementary material for: Efficacy and safety of CD19-specific CAR-T cell-based therapy in secondary central nervous system lymphoma
Source: Front Immunol. 2022 Aug 19;13:965224. doi: 10.3389/fimmu.2022.965224 (PMC9437350; doi:10.3389/fimmu.2022.965224)
Supplement: Supplementary file 1 [file DataSheet_1.doc]

[**supplementary**](javascript:;) [**data**](javascript:;)

**Table 1. Analysis of response of CNS involvement in patients with secondary CNSL according to baseline characteristics**

| **Characteristic** | **No. of patients** | **CR+PR of CNS** | **ORR** | **95% CI** | **P value** |
| --- | --- | --- | --- | --- | --- |
| **overall** | 15 | 11 | 0.733 | 0.480-0.987 |  |
| **Age** |  |  |  |  | 0.569 |
| <60 | 8 | 5 | 0.625 | 0.192-1.058 |  |
| ≥60 | 7 | 6 | 0.857 | 0.508-1.207 |  |
| **Gender** |  |  |  |  | 0.516 |
| M | 11 | 9 | 0.818 | 0.546-1.090 |  |
| F | 4 | 2 | 0.500 | -.419-1.419 |  |
| **aaIPI score** ^1^ |  |  |  |  | 0.604 |
| 0~2 | 6 | 5 | 0.833 | 0.405-1.262 |  |
| 3~4 | 9 | 6 | 0.667 | 0.282-1.051 |  |
| **Disease status** |  |  |  |  | 0.154 |
| Systemic + CNS | 12 | 10 | 0.833 | 0.586-1.081 |  |
| Isolated CNS | 3 | 1 | 0.333 | -1.101-1.768 |  |
| **Primary refractory or relapse** |  |  |  |  | 0.569 |
| Primary refractory | 8 | 5 | 0.625 | 0.192-1.058 |  |
| Relapse | 7 | 6 | 0.857 | 0.508-1.207 |  |
| **Cerebrospinal fluid infiltration** |  |  |  |  | 0.604 |
| Yes | 6 | 5 | 0.833 | 0.405-1.262 |  |
| No | 9 | 6 | 0.667 | 0.282-1.051 |  |
| **Types of CNS disease** |  |  |  |  | 1.000 |
| Single lesion | 8 | 6 | 0.750 | 0.363-1.137 |  |
| Multiple lesions | 7 | 5 | 0.714 | 0.263-1.166 |  |
| **Double - or triple - hit rearrangement** ^2^ |  |  |  |  | 0.516 |
| Yes | 3 | 2 | 1.000 | 1.000-1.000 |  |
| No | 12 | 9 | 0.667 | 0.354-0.980 |  |
| **P53 mutation** |  |  |  |  | 1.000 |
| Yes | 2 | 2 | 1.000 | 1.000-1.000 |  |
| No | 13 | 9 | 0.692 | 0.402-0.983 |  |
| **Prior Auto-HSCT** |  |  |  |  | 1.000 |
| Yes | 2 | 2 | 1.000 | 1.000-1.000 |  |
| No | 13 | 9 | 0.692 | 0.402-0.983 |  |
| **Bridging treatment** |  |  |  |  | 1.000 |
| Yes | 8 | 6 | 0.750 | 0.363-1.137 |  |
| No | 7 | 5 | 0.714 | 0.263-1.166 |  |
| **Cell dose** |  |  |  |  | 0.516 |
| 10^6^/kg | 11 | 9 | 0.818 | 0.546-1.090 |  |
| 10^7^/kg | 4 | 2 | 0.500 | -.419-1.419 |  |

### 1 aaIPI: age-adjusted international prognostic index; 2, Double - or triple - hit rearrangement：MYC and BCL2 and/or BCL6 Rearrangements;

**Table 2. Analysis of response in patients with secondary CNSL according to previous treatment regimens**

| **Characteristic** | **No. of patients** | **CR+PR of CNS** | **ORR** | **95% CI** | **P value** |
| --- | --- | --- | --- | --- | --- |
| **overall** | 15 | 11 | 0.733 | 0.480-0.987 |  |
| **intrathecal injection** |  |  |  |  |  |
| Yes | 10 | 7 | 0.700 | 0.354-1.046 |  |
| No | 5 | 4 | 0.800 | 0.245-1.355 |  |
| **high-dose methotrexate** |  |  |  |  |  |
| Yes | 5 | 4 | 0.800 | 0.245-1.355 |  |
| No | 10 | 7 | 0.700 | 0.354-1.046 |  |
| **Ibrutinib** |  |  |  |  |  |
| Yes | 7 | 5 | 0.714 | 0.263-1.166 |  |
| No | 8 | 6 | 0.750 | 0.363-1.137 |  |
| **Whole brain radiotherapy** |  |  |  |  |  |
| Yes | 4 | 2 | 0.500 | -.419-1.419 |  |
| No | 11 | 9 | 0.818 | 0.546-1.090 |  |
| **Lenalidomide** |  |  |  |  |  |
| Yes | 5 | 4 | 0.800 | 0.245-1.355 |  |
| No | 10 | 7 | 0.700 | 0.354-1.046 |  |

| **Table 3 Analysis of PFS and OS in patients with secondary CNSL according to baseline characteristics.** | | | | | |
| --- | --- | --- | --- | --- | --- |
| **Characteristic** | **No. of patients** | **Median PFS (m)** | **P value** | **Median OS (m)** | **P value** |
| **overall** | 15 | 4 |  | 9 |  |
| **Age** |  |  | 0.893 |  | 0.141 |
| <60 | 8 | 2 |  | 9 |  |
| ≥60 | 7 | 4 |  | 9 |  |
| **Gender** |  |  | 0.244 |  | 0.222 |
| M | 11 | 2 |  | 7 |  |
| F | 4 | 8 |  | 11 |  |
| **aaIPI score** ^1^ |  |  | 0.668 |  | 0.892 |
| 0~2 | 6 | 2 |  | 9 |  |
| 3~4 | 9 | 5 |  | 9 |  |
| **Cerebrospinal fluid infiltration** |  |  | 0.914 |  | 0.831 |
| Yes | 6 | 2 |  | 7 |  |
| No | 9 | 4 |  | 9 |  |
| **Types of CNS disease** |  |  | 0.807 |  | 0.992 |
| Single lesion | 8 | 4 |  | 7 |  |
| Multiple lesions | 7 | 2 |  | 9 |  |
| **Double - or triple - hit rearrangement** ^2^ |  |  | 0.432 |  | 0.259 |
| Yes | 3 | 8 |  | 11 |  |
| No | 12 | 2 |  | 7 |  |
| **P53 mutation** |  |  | 0.175 |  | 0.420 |
| Yes | 2 | 1 |  | 5 |  |
| No | 13 | 5 |  | 9 |  |
| **Prior Auto-HSCT** |  |  | 0.693 |  | 0.849 |
| Yes | 2 | 2 |  | 9 |  |
| No | 13 | 4 |  | 11 |  |
| **Bridging treatment** |  |  | 0.826 |  | 0.405 |
| Yes | 8 | 2 |  | 9 |  |
| No | 7 | 5 |  | 7 |  |
| **Cell dose** |  |  | 0.165 |  | 0.169 |
| 10^6^/kg | 11 | 5 |  | 11 |  |
| 10^7^/kg | 4 | 1 |  | 7 |  |

1，aaIPI: age-adjusted international prognostic index; 2, Double - or triple - hit rearrangement：MYC and BCL2 and/or BCL6 Rearrangements;

| **Table 4 Analysis of PFS and OS in patients with secondary CNSL according to previous treatment regimens** | | | | | |
| --- | --- | --- | --- | --- | --- |
| **Characteristic** | **No. of patients** | **Median PFS (m)** | **P value** | **Median OS (m)** | **P value** |
| **overall** | 15 | 4 |  | 9 |  |
| **intrathecal injection** |  |  | 0.686 |  | 0.652 |
| Yes | 10 | 2 |  | 11 |  |
| No | 5 | 4 |  | 9 |  |
| **high-dose methotrexate** |  |  | 0.758 |  | 0.798 |
| Yes | 5 | 5 |  | 7 |  |
| No | 10 | 2 |  | 9 |  |
| **Ibrutinib** |  |  | 0.737 |  | 0.729 |
| Yes | 7 | 2 |  | 9 |  |
| No | 8 | 4 |  | 7 |  |
| **Whole brain radiotherapy** |  |  | 0.628 |  | 0.595 |
| Yes | 4 | 8 |  | 11 |  |
| No | 11 | 2 |  | 7 |  |
| **Lenalidomide** |  |  | 0.641 |  | 0.813 |
| Yes | 5 | 2 |  | 9 |  |
| No | 10 | 4 |  | 7 |  |
